# Photosensing and Characterizing of the Pristine and In-, Sn-Doped Bi_2_Se_3_ Nanoplatelets Fabricated by Thermal V–S Process

**DOI:** 10.3390/nano11051352

**Published:** 2021-05-20

**Authors:** Chih-Chiang Wang, Fuh-Sheng Shieu, Han C. Shih

**Affiliations:** 1 Department of Materials Science and Engineering, National Chung Hsing University, Taichung 40227, Taiwan; wilbur0913@gmail.com; 2 Department of Chemical Engineering and Materials Science, Chinese Culture University, Taipei 11114, Taiwan

**Keywords:** Bi_2_Se_3_, nanoplatelets, defects, optical bandgap, photocurrent

## Abstract

Pristine, and In-, Sn-, and (In, Sn)-doped Bi_2_Se_3_ nanoplatelets synthesized on Al_2_O_3_(100) substrate by a vapor–solid mechanism in thermal CVD process via at 600 °C under 2 × 10^−2^ Torr. XRD and HRTEM reveal that In or Sn dopants had no effect on the crystal structure of the synthesized rhombohedral-Bi_2_Se_3_. FPA–FTIR reveals that the optical bandgap of doped Bi_2_Se_3_ was 26.3%, 34.1%, and 43.7% lower than pristine Bi_2_Se_3_. XRD, FESEM–EDS, Raman spectroscopy, and XPS confirm defects ($In^{3+}{}_{Bi^{3+}}$), ($In^{3+}{}_{V^{0}}$), ($Sn^{4+}{}_{Bi^{3+}}$), ($V^{0}{}_{Bi^{3+}}$), and ($Sn^{2+}{}_{Bi^{3+}}$). Photocurrent that was generated in (In,Sn)-doped Bi_2_Se_3_ under UV(8 W) and red (5 W) light revealed stable photocurrents of 5.20 × 10^−10^ and 0.35 × 10^−10^ A and high I_photo_/I_dark_ ratios of 30.7 and 52.2. The rise and fall times of the photocurrent under UV light were 4.1 × 10^−2^ and 6.6 × 10^−2^ s. Under UV light, (In,Sn)-dopedBi_2_Se_3_ had 15.3% longer photocurrent decay time and 22.6% shorter rise time than pristine Bi_2_Se_3_, indicating that (In,Sn)-doped Bi_2_Se_3_ exhibited good surface conduction and greater photosensitivity. These results suggest that In, Sn, or both dopants enhance photodetection of pristine Bi_2_Se_3_ under UV and red light. The findings also suggest that type of defect is a more important factor than optical bandgap in determining photo-detection sensitivity. (In,Sn)-doped Bi_2_Se_3_ has greater potential than undoped Bi_2_Se_3_ for use in UV and red-light photodetectors.

## 1. Introduction

Bi_2_Se_3_ is a well-known second-generation topological insulator (TI) with a narrow bandgap of 0.35 eV and a rhombohedral crystal structure [1]. Se vacancies ($v_{\mathrm{Se}}$), which act as electron donors, are the main defects in the Bi_2_Se_3_ structure, making it an n-type topological insulator [2]. The crystalline Bi_2_Se_3_ is composed of layered structures; each layer consists of five stacked monoatomic layers, as in Se-Bi-Se’-Bi-Se, and is thus known as a quintuple layer (QL) [3]. Covalent bonds dominate the QL [4], whereas Van der Waals’ forces dominate between QLs; hence, the dopants can be adequately intercalated among them [5]. A TI has an insulating bulk state and a topologically protected gapless surface state in three dimensions and an edge state in two dimensions, owing to spin-orbital coupling (SOC) and time-reversal symmetry (TRS) [6,7]. Both SOC and TRS suppress backscattering and reduce the sensitivity to surface impurities or defects when electrons are transported on the surface of a TI. These gapless states thus lead to a high electronic conductivity [8,9] and the following features: (1) photon-like electrons, (2) low power dissipation, (3) spin-polarized electrons, and (4) the quantum spin Hall effect [10,11,12,13]. Owing to TIs’ unique electronic properties, they have many potential applications, including photodetectors [14], lasers [15], gas sensors [16], spintronic devices [17], magnetoelectronic devices [18], quantum computers [19], and topological superconductors [20]. Several methods are commonly used to synthesize TIs; they include chemical vapor deposition [21], mechanical exfoliation [22], solvothermal synthesis [23], molecular beam epitaxy [24], atomic layer epitaxy [25], metal–organic chemical vapor deposition [26], pulsed laser deposition [27], magnetron sputtering [28], and the Bridgeman method [29].

Broadband photodetectors are made of potential materials, whose conductivity can be changed by incident light such as UV, visible, or IR light; they have photoabsorbance over a wide range of wavelengths, high photosensitivity, high carrier mobility, high conservation efficiency, operability at a low voltage, and long operational stability [30,31]. Photon–electron transfer is the primary detection mechanism of photodetectors [32]. Thus, photodetectors can be used as potential materials in optical information communication, imaging detection, and biodetectors [33,34]. As required for use in photodetectors, topological insulating Bi_2_Se_3_ is a potential material and has fascinating optoelectronic properties, such as a tunable surface bandgap, a polarization-sensitive photocurrent, and thickness-dependent optical absorption [35]. Bulk Bi_2_Se_3_ has a narrow bandgap of 0.35 eV and therefore a wide range of absorption wavelengths. However, its surface transportation can be suppressed by the free carriers that are presented in its bulk state. Shrinking to the nanoscale, as for nanoplates, nanowires, nanoribbons, and thin films, it suppresses the contribution of the bulk state to reduce the surface transportation, strengthening its surface transportation by increasing the surface-to-volume ratio [7,9]. Supplementary Table S1 lists Bi_2_Se_3_-based photodetectors.

The optical bandgap of Bi_2_Se_3_ depends on the synthesizing conditions. Pejova et al. reported that chemically deposited Bi_2_Se_3_ thin films have an optical bandgap energy of 2.3 eV [36]; Pramanik et al. fabricated Bi_2_Se_3_ with optical bandgaps of 1.03 and 1.15 eV by chemical deposition [37]. Garcia et al. reported that chemically prepared Bi_2_Se_3_ films had optical bandgaps of 1.41–1.7 eV and that postannealing (200 °C) Bi_2_Se_3_ films had bandgaps of 1.06–1.57 eV [38]. Manjulavalli et al. found that Bi_2_Se_3_ films that were prepared by thermal evaporation had an optical bandgap of 0.825 eV, and annealed films had a gap of 0.61 eV [39]. Augustine et al. reported that Bi_2_Se_3_ films that were fabricated by thermal evaporation had an optical bandgap of 0.67 eV [40]. Alemi et al. synthesized Bi_2_Se_3_ nanoplatelets, which had a bandgap of 2.95 eV, by a hydrothermal process [41]. These results reveal that the absorption range of Bi_2_Se_3_ can be narrowed, affecting its photo-detective efficiency. According to the relevant literature, doping can modify the optical bandgaps of Bi_2_Se_3_ nanostructures. Supplementary Table S2 presents the variations of bandgap energy of Bi_2_Se_3_ that is doped with various dopants.

Bismuth (Bi, melting point = 271.4 °C), selenium (Se, melting point = 220 °C), indium (In, melting point = 156.6 °C), and tin (Sn, melting point = 231.9 °C) have similar melting points. The covalent radii of Bi (~148 pm), In (~142 pm), and Sn (~139 pm) are similar. The thermal–CVD process was used to synthesize pristine Bi_2_Se_3_ nanoplatelets on Al_2_O_3_(100) substrates by the vapor–solid growth mechanism at 600 °C under 1.2 × 10^−2^ Torr. The photocurrents in In-, Sn-, and (In, Sn)-doped Bi_2_Se_3_ nanoplatelets have not been studied. Dopants In and Sn are used herein to fabricate In-, Sn-, and (In, Sn)-doped Bi_2_Se_3_ nanoplatelets. The types of defects and effects of In and Sn dopants on the Bi_2_Se_3_ crystal structure were investigated by XRD, HRTEM, XPS, and Raman spectroscopy. The obtained optical bandgaps were estimated using an FPA–FTIR spectrometer. The photosensitivities were systematically obtained by measuring the photocurrents under UV and red light.

## 2. Materials and Methods

### 2.1. Synthesis of Pristine and In-, Sn-Doped Bi_2_Se_3_ Nanoplatelets

Supplementary Figure S1 schematically depicts the synthetic system. Pristine Bi_2_Se_3_ nanoplatelets were synthesized on Al_2_O_3_(100) substrate (0.5 × 0.5 mm^2^) by the catalyst-free V–S mechanism using a thermally activated process in a quartz tube furnace. A 0.2 g mixture of precursor powders of high-purity 0.1 g Bi (Merck, 99%, 4.78 × 10^−4^ mole, Darmstadt, Germany) and 0.1 g Se (Alfa Aesar, 99%, 1.27 × 10^−3^ mole, Ward Hill, MA, USA) was placed on an alumina boat in the heating zone at the center of a quartz tube and heated at a rate of 25 °C/min under 1.2 × 10^−2^ Torr to 600 °C, which was maintained for 60 min. Al_2_O_3_(100) substrate was placed upstream in the quartz tube at about 150 °C, about 21 cm away from the alumina boat. The pristine Bi_2_Se_3_ nanoplatelets thus formed were then deposited on the Al_2_O_3_(100) substrate. After the 60 min deposition process, the deposition system was subsequently cooled to room temperature. The starting materials of In-doped Bi_2_Se_3_ were 0.1 g Bi, 0.1 g Se, and 25 mg of high-quality In as dopant (Alfa Aesar, 99.99%, 2.17 × 10^−4^ mole, USA); those of Sn-doped Bi_2_Se_3_ included 25 mg Sn as dopant (Alfa Aesar, 99.8%, 2.11 × 10^−4^ mole, USA); that of (In, Sn)-doped Bi_2_Se_3_ included 12.5 mg co-dopants In (1.09 × 10^−4^ mole) and 12.5 mg Sn (1.05 × 10^−4^ mole). The In-, Sn-, and (In, Sn)-dopedBi_2_Se_3_ nanoplatelets were synthesized in the same manner as the pristine Bi_2_Se_3_ nanoplatelets.

### 2.2. Characterization of Nanoplatelets

The phase and crystal structures of the undoped, as well as the In-, Sn-, and (In, Sn)-doped Bi_2_Se_3_ nanoplatelets were determined using a mass absorption coefficient glancing incident X-ray diffractometer with an incidence angle of 0.5° (λ = 0.154 nm, 30 A, 40 kV, Bruker D2 PHASER) and a high-resolution transmission electron microscope (JEOL, JEM-3000 F, Tokyo, Japan). The chemical binding energies and vibration modes of the chemical bonds were obtained using an X-ray photoelectron spectroscope (XPS, Perkin-Elmer model PHI1600 system, Waltham, MA, USA) and a Raman spectroscope (3D Nanometer Scale Raman PL Microspectrometer, Tokyo Instruments, Inc., Tokyo, Japan) with a semiconductor laser at an excitation wavelength of 633 nm. The surface morphology and EDS spectra were obtained using FESEM (ZEISS ULTRA PLUS, Carl Zeiss Microscopy GmbH, Oberkochen, Germany). The optical absorbance values were recorded using a focal plane array–FTIR spectrometer (FPA–FTIR, Bruker Vertex 70V, Hyperion 3000, 64 × 64 MCT Focal Plane Array, Bruker Optik GmbH, Ettlingen, Germany).

### 2.3. Photocurrent Analysis

Photocurrents were measured using a semiconductor I–V property analyzer (Keysight B2901A Precision Source/Measure Unit 100 fA, Keysight Technologies, Santa Rosa, CA, USA) under irradiations of UV or the red light at atmospheric pressure and room temperature, while the bias voltage was kept at 0 V during the photocurrent measuring. The irradiation sources were 30 cm long UV (8 W, λ = 365 nm) and red (5 W, λ = 700–900 nm) LED lamps. The distance between each lamp and the sample was 20 cm. Supplementary Figure S2 schematically depicts the photocurrent measuring system. The sample was placed in a closed box/darkroom to eliminate any effect of ambient light. The silver paste was dropped and deposited onto the surface of each sample of the nanoplatelets and connected to the photocurrent analyzer using copper wires. The photocurrent of each sample was measured in five runs; in each run, the light was on for 10 s and off for 10 s.

## 3. Results

### 3.1. XRD Analysis

**Figure 1:**
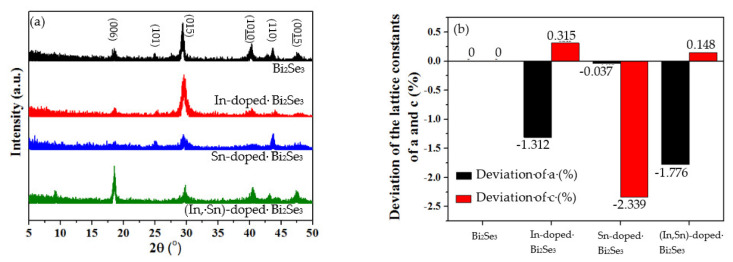
(**a**) XRD patterns and (**b**) deviations of the lattice constants a and c of pristine, and In-, Sn-, and (In, Sn)-doped Bi_2_Se_3_ nanoplatelets.

Figure 1a presents the XRD patterns of the pristine and In-, Sn-, and (In, Sn)-doped Bi_2_Se_3_ nanoplatelets. These nanoplatelets have the typical rhombohedral Bi_2_Se_3_ structure (JCPDS 89-2008). Table 1 presents the lattice constants a, b, c, and the c/a ratio. The lattice constants are calculated as the formula $\frac{1}{d_{\left(hkl\right)}^{2}}=\left[\frac{4}{3}\left(h^{2}+k^{2}+hk\right)+l^{2}{\left(\frac{a}{c}\right)}^{2}\right]\frac{1}{a^{2}}$, where *h*, *k*, and *l* are the Miller indices, and a and c are the lattice constants. For the In-, Sn-, and (In, Sn)-doped Bi_2_Se_3_ nanoplatelets, lattice constant a deviates by −1.312, −0.037, and −1.776%, respectively, and lattice constant c deviates by 0.315, −2.339, and 0.148%, respectively, from their values for the undoped nanoplatelets. The decreases in a are attributed to the substitution of Bi (covalent radius: 148 ± 4 pm) rather than Se (covalent radius: 120 ± 4 pm) by the In (covalent radius: 142 ± 5 pm) or Sn (covalent radius: 139 ± 4 pm) dopant. The lowering of c by doping with Sn is attributed to the substitution of Bi by Sn [42]. The bond length of Bi-Se is 2.86/3.05 Å, and the gap between each QL is 2.62 Å [43]; therefore, the increase in c upon doping with In or (In, Sn) is attributable to the intercalation of In atoms between the QLs [44]. The decrease and increase of the lattice constants a and c, respectively, imply that the In and Sn dopants affected the crystallinity of the Bi_2_Se_3_ nanoplatelets. Defects are formed by In at Bi lattice sites ($In_{Bi}$), Sn at Bi lattice sites ($Sn_{Bi}$), and In in vacancies ($In_{V}$).

### 3.2. Structural and Surface Morphology Analyses

**Figure 2:**
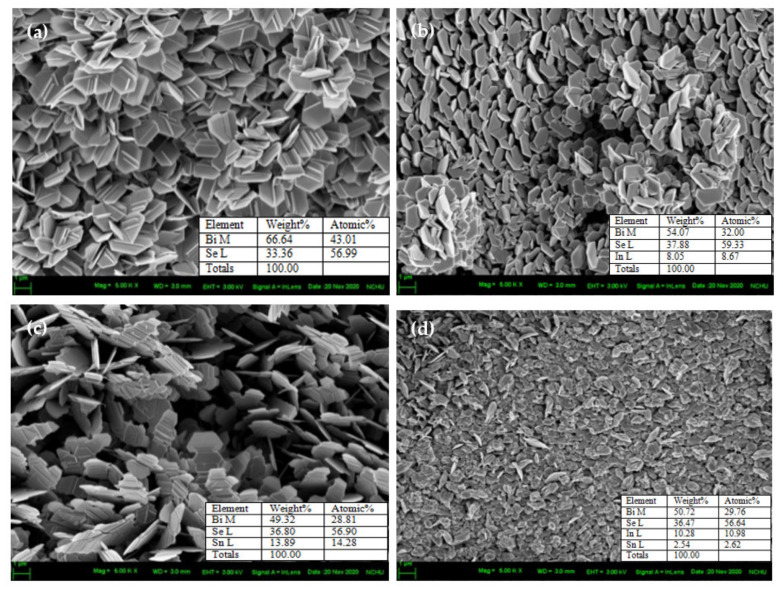
FESEM images and EDS results of (**a**) pristine, (**b**) In-, (**c**) Sn-, and (**d**) (In, Sn)-doped Bi_2_Se_3_ nanoplatelets.

Figure 2a–d and the respective thickness of Figure S3a–d show the cross-sectional and the plane-view images of pristine and In-, Sn-, and (In,Sn)-doped Bi_2_Se_3_ nanoplatelets, respectively. The overall thickness of the samples of pristine and In-, Sn-, and (In, Sn)-doped Bi_2_Se_3_ nanoplatelets are, respectively, of the 3.391, 0.859, 1.066, and 0.563 μm, as shown in Figure S3a–d. The hexagonal Bi_2_Se_3_ nanoplatelets (Figure 2a and Figure S3a) appear in similar morphologies after the doping.

The In- (Figure 2b and Figure S3b) and (In,Sn)-doped Bi_2_Se_3_ (Figure 2d and Figure S3d) reveal less well-defined hexagonal structures; however, the Sn-doped Bi_2_Se_3_ (Figure 2c and Figure S3c) exhibits a very well-defined hexagonal structure. On average, the Bi_2_Se_3_ nanoplatelets are unequivocally hexagonal-like in shape, typical of the rhombohedral structure. The average thickness (40 nanoplatelets) and average diameter (40 nanoplatelets) of the pristine and In-, Sn-, and (In, Sn)-doped Bi_2_Se_3_ nanoplatelets are, respectively, listed in Table 2.

**Figure 3:**
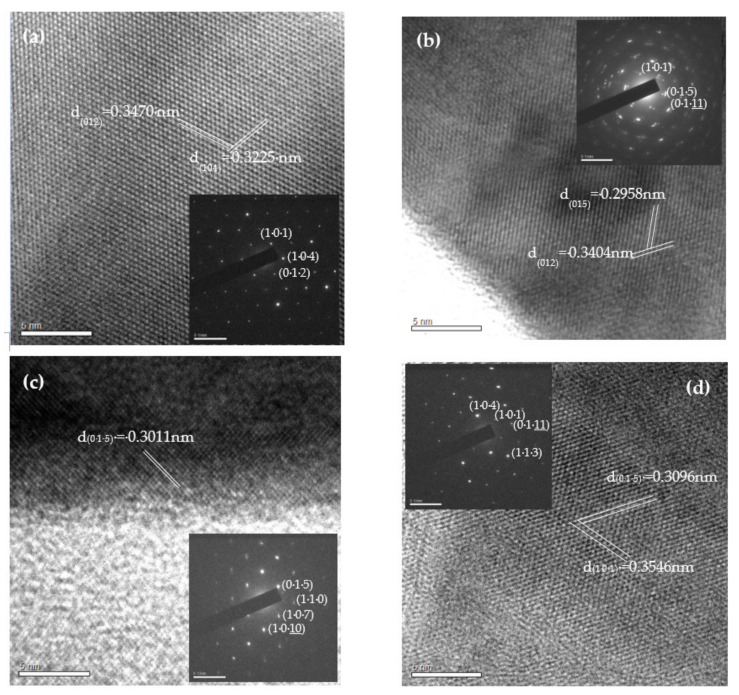
HRTEM images and SAD patterns of (**a**) Bi_2_Se_3_, (**b**) In-, (**c**) Sn-, and (**d**) (In, Sn)-doped Bi_2_Se_3_ nanoplatelets.

Table 2 shows EDS results for Bi, Se, In, and Sn, which reveal that the ratio Bi:Se increases upon the addition of dopants. This result is attributable to the substitution of Bi by In and/or Sn dopants. Under the Se-rich condition (mole ratio, Bi/Se = 0.755) in this work, the formation energy of $V_{Se}$ defects increases from 1.14 to 2.16 eV, and that of $V_{Bi}$ decreases from 4.13 to 2.60 eV [42]; Bi is thus determined to be substituted by In and/or Sn dopants, consistent with the XRD results. Figure 3 presents the HRTEM images and SAD patterns of pristine (Figure 3a), and In- (Figure 3b), Sn- (Figure 3c), and (In, Sn)-doped Bi_2_Se_3_ nanoplatelets (Figure 3d). Table 3 provides d-spacings and diffraction planes. These results are consistent with the rhombohedral Bi_2_Se_3_ structure and confirm that the dopants, such as In and Sn, have no effect on the crystal structure of Bi_2_Se_3_.

### 3.3. XPS Analysis

**Figure 4:**
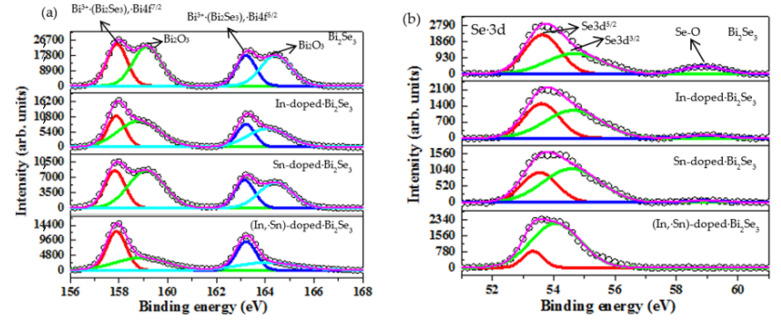

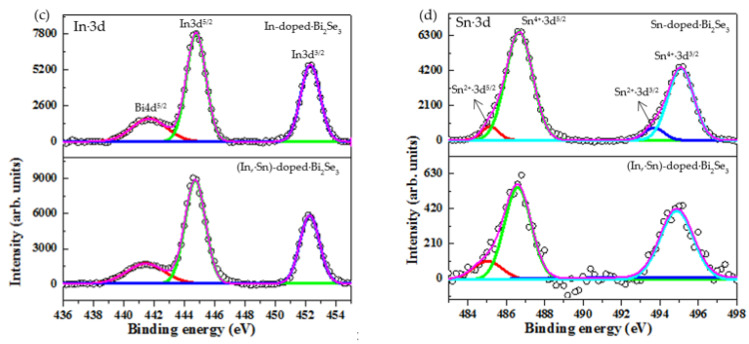
XPS spectra of (**a**) Bi 4f, (**b**) Se 3d, (**c**) In 3d, and (**d**) Sn 3d in pristine, and Sn-, and (In, Sn)-doped Bi_2_Se_3_ nanoplatelets.

Figure 4 presents the XPS results for Bi 4f, Se 3d, In 3d, and Sn 3d. Figure 4a shows the binding energy of Bi 4f. Peaks at 157.9 and 163.2 eV are attributed to the Bi 4f^7/2^ and Bi 4f^5/2^ orbitals in the Bi_2_Se_3_ phase [45]. Binding energies 159.1 and 164.3 eV are associated with the Bi 4f^7/2^ and Bi 4f^5/2^ orbitals in the Bi_2_O_3_ phase [46,47]. Figure 4b presents the XPS spectra of Se 3d^5/2^ and Se 3d^3/2^ and the binding energies of 53.6 and 54.6 eV are associated with the Bi_2_Se_3_ phase [45]. The peak at 58.9 eV is attributed to the SeO_2_ phase [48,49]. The samples are stored in the ambient environment, causing the Bi_2_O_3_ and SeO_2_ phases to form on the surface of the nanoplatelets. These results confirm the formation of the Bi_2_Se_3_ phase by the thermal V–S mechanism. Figure 4c displays the binding energies of the In 3d^5/2^ and In 3d^3/2^ orbitals, 444.7 and 452.3 eV, associated with the In-Se bond [50]. This finding reveals that In^3+^ was doped into the Bi_2_Se_3_ structure. The typical Bi_2_Se_3_ structure consists of a stack of several quintuple layers (QLs). Each QL comprises Se-Bi-Se’-Bi-Se. In^3+^ has two possible positions as a dopant: (1) In^3+^ may substitute at the Bi^3+^ lattice sites, producing the neutral defect ($In^{3+}{}_{Bi^{3+}}$) and (2) In^3+^ intercalates between pairs of QLs, ind icating the possible formation of potentially forming the donor defect ($In^{3+}{}_{V^{0}}$), where V is the vacancy in the Van der Waals gap. Therefore, In-Se bonds form inside the Bi_2_Se_3_ structure or between QLs. The peak at 441.5 eV is ascribed to the Bi^3+^ 4d^5/2^ orbital [51]. Figure 4d presents XPS spectra of Sn 3d. Peaks at 485.1 and 493.7 eV are associated with the Sn^2+^ 3d^5/2^ and Sn^2+^ 3d^3/2^ orbitals, respectively, of the SnSe phase [52]. Peaks at 486.6 and 495.1 eV are associated with the Sn^4+^ 3d^5/2^ and Sn^4+^ 3d^3/2^ orbitals of the SnSe_2_ phase [53]. Accordingly, the Sn dopants substitute at some of the Bi lattice sites within the Bi_2_Se_3_ crystal structure and bond with Se to form Sn-Se bonds. The integral area of the Sn^4+^ in the XPS spectrum exceeds that of Sn^2+^ (as shown in Figure 4d), implying that the Sn^4+^ contents are higher than the Sn^2+^ content. The XRD results reveal that the defect ($Sn_{Bi}$) is formed in the Bi_2_Se_3_ structure during the thermal V–S process. The concentration of the donor defects ($Sn^{4+}{}_{Bi^{3+}}$) should be higher than those of the acceptor defects ($Sn^{2+}{}_{Bi^{3+}}$).

### 3.4. Raman Spectra

**Figure 5:**
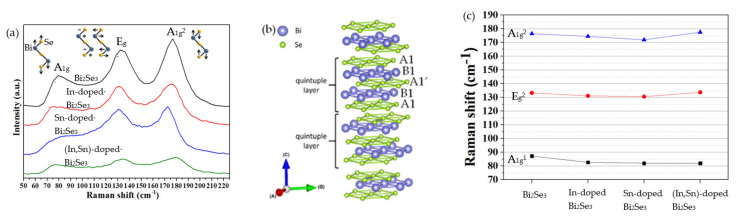
(**a**) Raman spectra of pristine, and In-, Sn-, and (In, Sn)-doped Bi_2_Se_3_ nanoplatelets; the inset is the schematic vibration modes of A_1g_^1^, E_g_^2^, and A_1g_^2^ [56,58]. (**b**) Schematic layered structure of Bi_2_Se_3_ [55]. (**c**) Variations of Raman shift at A_1g_^1^, E_g_^2^, and A_1g_^2^ with different dopants.

Figure 5a presents the typical Raman active mode at A_1g_^1^, E_g_^2^, and A_1g_^2^ of the rhombohedral Bi_2_Se_3_ structure [6,54]. No other peaks besides Bi-Se vibrational modes are observed; hence, the dopant does not change the crystal structure of the nanoplatelets or form a second-phase compound, consistent with the XRD results. The formation of Bi_2_Se_3_ nanoplatelets is thus confirmed.

The typical structure of Bi_2_Se_3_ is layered; each layer comprises five monoatomic planes and is, therefore, a quintuple layer (QL). The QL is denoted as A1-B1-A1’-B1-A1, as shown in Figure 5b [55]. A1 and A1’ are the Se atoms; B1 is the Bi atom. Covalent bonds dominate the binding within each QL; Van der Waals’ forces dominate the bonds between QLs [4,5]. The inset in Figure 5a schematically depicts the Raman peaks of A_1g_^1^, E_g_^2^, and A_1g_^2^ [56]. A_1g_ is a symmetric out-of-plane stretching mode associated with the vibration of A1-B1 atoms in the same (A_1g_^1^ mode) or the opposite (A_1g_^2^ mode) direction. A_1g_^2^ has a shorter atomic displacement than A_1g_^1^. Therefore, the A_1g_^2^ mode has higher phonon energy than the A_1g_^1^ mode [56]. E_g_^2^ is a symmetric in-plane bending mode and shearing the upper two layers of A1-B1 atoms that vibrate in the opposite direction increasing the atomic displacement to a value greater than that in the A_1g_^2^ mode but smaller than that in the A_1g_^1^ mode. Thus, the E_g_^2^ mode has a phonon energy between those of the A_1g_^2^ and A_1g_^1^ modes [56].

Figure 5c shows the variations in characteristic Raman peaks at A_1g_^1^, E_g_^2^, and A_1g_^2^ with the species of dopant. A comparison with pristine Bi_2_Se_3_ nanoplatelets reveals that both In and Sn dopants cause a redshift of the peaks of A_1g_^1^, E_g_^2^, and A_1g_^2^, whereas (In, Sn) co-dopants do not shift the A_1g_^2^ or E_g_^2^ peak but do cause a redshift in the A_1g_^1^ peak. Table 4 presents the Raman peaks of pristine and doped Bi_2_Se_3_ nanoplatelets, showing the redshifts with the various dopants. The redshifts of the Raman peaks are frequently suggested to involve the heavier atomic weight and/or high-electronegativity dopant to be doped in [57]. The atomic weights of Bi, Se, In, and Sn are 209.0, 78.76, 114.8, and 118.7 (g/mole), and their electronegativities are 2.02, 2.55, 1.96, and 1.78, respectively. The Raman peaks of the doped Bi_2_Se_3_ nanoplatelets thus exhibit a redshift. As shown in Table 4, E_g_^2^, A_1g_^2,^ and especially A_1g_^1^ peaks are significantly redshifted by the addition of different dopants. The redshift is attributed to the substitution of Bi with dopant In or Sn, which has less weight and a lower electronegativity.

### 3.5. Photocurrent under UV and Red Light

#### 3.5.1. Analysis under UV and Red Illumination

**Figure 6:**
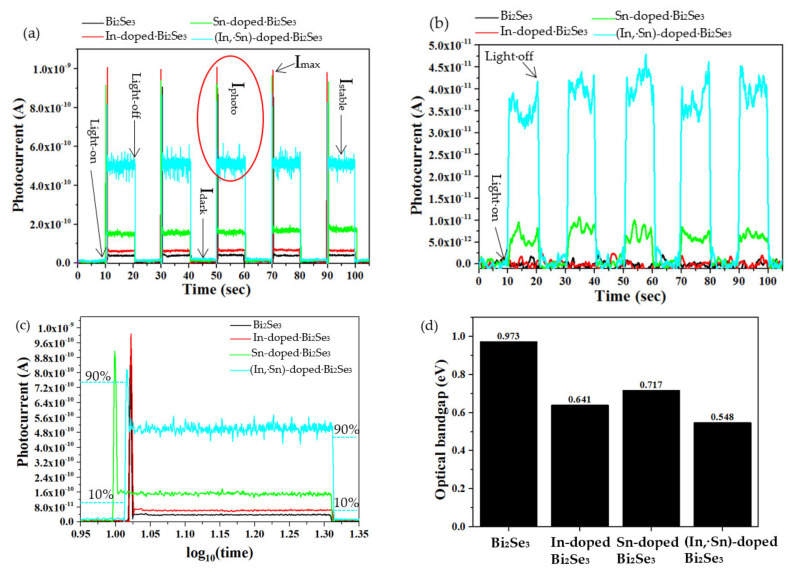
Photocurrent plots of pristine, and In-, Sn-, and (In, Sn)-doped Bi_2_Se_3_ nanoplatelets under (**a**) UV (8 W) and (**b**) red light (5 W). (**c**) The variations of the log_10_(time) vs. the photocurrents between the pristine, and In-, Sn-, and (In,Sn)- doped Bi_2_Se_3_ nanoplatelets in the first run of the light-on/light-off cycle. (**d**) The estimated optical bandgap energies.

Figure 6a shows the photocurrents in undoped, as well as In-, Sn-, and (In, Sn)-doped Bi_2_Se_3_ nanoplatelets under UV light. All samples pass a photocurrent (I_photo_) that increases rapidly from I_dark_ (~0.62 × 10^−11^–1.65 × 10^−11^ A) to the maximum I_max_ (~8 × 10^−10^–1 × 10^−9^ A) and then suddenly falls to a stable value (I_stable_) when the light is turned on. I_stable_ is clearly independent of the exposure time and, for the undoped, and In-, Sn-, and (In, Sn)-doped Bi_2_Se_3_ nanoplatelets, it is 0.40 × 10^−10^, 0.65 × 10^−10^, 1.60 × 10^−10^, and 5.20 × 10^−10^ A, respectively, indicating that the dopants can increase the photocurrent of the pristine Bi_2_Se_3_ nanoplatelets. In particular, the co-dopants In and Sn increase it by a factor of more than 13 to, for example, 5.20 × 10^−10^ A. The I_photo_/I_dark_ ratios in the undoped, and In-, Sn-, and (In, Sn)-doped Bi_2_Se_3_ nanoplatelets are, respectively, estimated as 7.66, 9.29, 15.8, and 30.7, as shown in Figure S4a and listed in Table 5, implying that the co-dopants of In and Sn enhance the photoresponsibility of the Bi_2_Se_3_ nanoplatelets 4.01 times, which is higher than the pristine one. The decay of the current ΔI_decay_ (I_max_–I_stable_) in the undoped, as well as In-, Sn-, and (In, Sn)-doped Bi_2_Se_3_ nanoplatelets, is 8.33 × 10^−10^, 9.33 × 10^−10^, 7.76 × 10^−10^, and 3.21 × 10^−10^ A, respectively. A smaller ΔI_decay_ is attributed to a longer electron lifetime and a higher concentration of electrons. Both the rise time (τ_r_) and fall time (τ_f_) are taken by the photocurrent to rise or to fall from 10% to 90% or from 90% to 10%, respectively, of its maximum photocurrent value, as an example of the (In,Sn)-doped Bi_2_Se_3_ in Figure 6c. The average τ_r_ for the undoped, and In-, Sn-, and (In, Sn)-doped Bi_2_Se_3_ nanoplatelets are, respectively, evaluated as 0.053, 0.051, 0.044, and 0.041 sec; the average τ_f_ are 0.049, 0.050, 0.054, and 0.066 sec. A shorter τ_r_ is attributable to the higher photosensitivity. The decay time (t_decay_) is taken from the I_max_ to I_stable_, which depends on the recombination rate of the photo-induced electrons and holes [59]. The average t_decay_ of the undoped, and In-, Sn-, and (In, Sn)-doped Bi_2_Se_3_ nanoplatelets are estimated as 0.091, 0.097, 0.099, and 0.105 sec. A longer t_decay_ corresponds to slower recombination. Therefore, the co-dopants of In and Sn suppress the recombination rate of the photoinduced electrons and holes in the pristine Bi_2_Se_3_ nanoplatelets. The detailed variations of the log_10_(time) versus the photocurrents, which are recorded in the first run of the light-on/light-off cycle, between the pristine, and In-, Sn-, and (In,Sn)-doped Bi_2_Se_3_ nanoplatelets are presented in Figure 6c. Table 5 presents relevant details. These results suggest that the dopants, and especially the co-dopants In and Sn, in Bi_2_Se_3_ nanoplatelets, have various favorable effects, which are (1) extending the electron lifetime, (2) increasing the electron concentration, (3) promoting surface electronic transportation, and (4) improving the photo-sensitivity of the Bi_2_Se_3_ nanoplatelets.

Figure 6b presents variations of the photocurrent of the undoped, and In-, Sn-, and (In, Sn)-doped Bi_2_Se_3_ nanoplatelets under red light. Both undoped and In-doped Bi_2_Se_3_ generate no photocurrent, whereas Sn- and (In, Sn)-doped Bi_2_Se_3_ generate a photocurrent of 0.5 × 10^−10^ and 3.5 × 10^−10^ A when the red light is turned on. These results reveal that the photosensitivity of Bi_2_Se_3_ nanoplatelets to a red light is greatly improved by the dopants, and especially by the co-dopants In and Sn. The I_photo_/I_dark_ ratios of the undoped, and In-, Sn-, and (In, Sn)-doped Bi_2_Se_3_ nanoplatelets are, respectively, estimated as 1, 1, 20.9, and 52.2, as shown in Figure S4b. The co-dopants of In and Sn enhance the Bi_2_Se_3_ nanoplatelets 52.2 times higher than that of the undoped one.

#### 3.5.2. Effects of the Defect Structure and Optical Bandgap on Photocurrent

Figure 6d shows that the optical bandgaps, estimated from the Tauc plot [60], of the undoped, as well as In-, Sn-, and (In, Sn)-doped Bi_2_Se_3_ nanoplatelets, are 0.973, 0.641, 0.717, and 0.548 eV, respectively. These bandgaps are estimated by the following equation of ${\left(\alpha h\nu \right)}^{n}=A\left(h\nu -E_{g}\right)$, where α is the absorption coefficient, h is the Planck’s constant, ν is the light frequency, n is the characteristic coefficient of materials, A is a constant, and $E_{g}$ is the bandgap. For the direct bandgap of the Bi_2_Se_3_, n is 2. Their absorbance spectra, which were recorded by FPA–FTIR, are shown in Figure S5. Each individual Tauc plot of the pristine, In-, Sn-, and (In,Sn)-doped Bi_2_Se_3_ nanoplatelets is demonstrated in Figure S6. These small bandgaps show that the incident UV (~3.4 eV) and red (~1.37–1.77 eV) light easily generate photo-induced electrons and holes. A photocurrent is therefore detectable in all of the samples of interest. However, the variously doped Bi_2_Se_3_ nanoplatelets exhibit significantly different photocurrents. Defect structures are the dominant factor that affects the photocurrent. Possible defects are ($In^{3+}{}_{Bi^{3+}}$), ($In^{3+}{}_{V^{0}}$), ($Sn^{4+}{}_{Bi^{3+}}$), ($V^{0}{}_{Bi^{3+}}$), and ($Sn^{2+}{}_{Bi^{3+}}$), where ($In^{3+}{}_{Bi^{3+}}$) a neutral defect; ($In^{3+}{}_{V^{0}}$) and ($Sn^{4+}{}_{Bi^{3+}}$) are donor defects and supply additional electrons; and ($V^{0}{}_{Bi^{3+}}$) and ($Sn^{2+}{}_{Bi^{3+}}$) are acceptor defects and supply additional holes. ($V^{0}{}_{Bi^{3+}}$) is the main defect in undoped Bi_2_Se_3_, whose photocurrent under UV or red light is, therefore, the lowest or ~0 A. ($In^{3+}{}_{Bi^{3+}}$) and ($In^{3+}{}_{V^{0}}$) instead of ($V^{0}{}_{Bi^{3+}}$) are the main defects in In-doped Bi_2_Se_3_, and therefore, a photocurrent under UV is detected therein. However, no photocurrent is detected under red light, owing to the lower electron concentration and faster recombination of electrons and holes. ($Sn^{4+}{}_{Bi^{3+}}$) rather than ($Sn^{2+}{}_{Bi^{3+}}$) and ($V^{0}{}_{Bi^{3+}}$) is the main defect in Sn-doped Bi_2_Se_3_ nanoplatelets; hence, more electrons than undoped and In-doped Bi_2_Se_3_ nanoplatelets can be supplied and generate a high photocurrent under UV or red light. Accordingly, Sn-doped Bi_2_Se_3_ nanoplatelets exhibit a higher photocurrent than undoped and In-doped Bi_2_Se_3_ nanoplatelets. ($V^{0}{}_{Bi^{3+}}$) and ($Sn^{2+}{}_{Bi^{3+}}$) are not the main defects. Rather, ($In^{3+}{}_{V^{0}}$), ($Sn^{4+}{}_{Bi^{3+}}$), and ($In^{3+}{}_{Bi^{3+}}$) are the main defects in (In, Sn)-doped Bi_2_Se_3_ nanoplatelets. ($In^{3+}{}_{V^{0}}$) and ($Sn^{4+}{}_{Bi^{3+}}$) can supply more additional electrons than undoped, as well as In- and Sn-doped Bi_2_Se_3_ nanoplatelets, to generate the highest photocurrent. Therefore, the (In, Sn)-doped Bi_2_Se_3_ nanoplatelets have the highest photocurrents under UV and red light than do undoped, as well as In-, and Sn-doped Bi_2_Se_3_ nanoplatelets. Based on the above discussion, the reduced optical bandgap of the doped Bi_2_Se_3_ nanoplatelets is a minor factor that affects the photocurrent. The type of defect that is generated by doping has a greater effect on the photodetection sensitivity than the corresponding reduction of the optical bandgap.

## 4. Conclusions

(In, Sn)-doped Bi_2_Se_3_ nanoplatelets under UV (8 W) and red light (5 W) have a higher I_photo_/I_dark_ ratio of 30.7 and 52.2 and a stable photocurrent of 5.20 × 10^−10^ and 0.35 × 10^−10^ A, respectively, higher than that of the undoped Bi_2_Se_3_ nanoplatelets (UV light: 7.66, 0.4 × 10^−10^ A; red light: 1, 2.38 × 10^−1^^3^ A). (In, Sn)-doped Bi_2_Se_3_ nanoplatelets have a shorter photocurrent rise time (0.041 s) and a longer decay time (0.105 s) than undoped Bi_2_Se_3_ nanoplatelets (0.053 and 0.091 s) by about 22.6% and 15.3%, respectively. These results suggest that photodetection under UV and red light by pristine Bi_2_Se_3_ nanoplatelets can be improved by doping with In and Sn. The optical bandgap of pristine Bi_2_Se_3_ nanoplatelets is 0.973 eV; it can be reduced to 0.641, 0.717, and 0.548 eV, corresponding to reductions of 34.1%, 26.3%, and 43.7% by doping with In, Sn, and both In and Sn. Based on XRD, XPS, FESEM–EDS, and Raman spectra, ($In^{3+}{}_{V^{0}}$), ($Sn^{4+}{}_{Bi^{3+}}$), ($In^{3+}{}_{Bi^{3+}}$), ($V^{0}{}_{Bi^{3+}}$), and ($Sn^{2+}{}_{Bi^{3+}}$) were formed during the synthesis of nanoplatelets, and structural defects ($In^{3+}{}_{V^{0}}$) and ($Sn^{4+}{}_{Bi^{3+}}$) significantly improved the photocurrent of (In, Sn)-doped Bi_2_Se_3_ nanoplatelets under UV and red light. This work also reveals that In or Sn dopant has no effect on the crystal structure of rhombohedral Bi_2_Se_3_. These results suggest that the photodetection sensitivity of Bi_2_Se_3_ nanoplatelets is dominated by the defect structures that are generated by doping, as well as by the consequent reduction of optical bandgap energy. The co-dopants In and Sn further enhance the ability of Bi_2_Se_3_ nanoplatelets to respond to UV and red light.

## Figures and Tables

**Table 1:** Lattice constants a and c, c/a ratios of pristine, and In-, Sn-, and (In, Sn)-doped Bi_2_Se_3_ nanoplatelets.

Sample	Lattice Constant		c/a Ratio	Concentration (×10^−4^ Mole)			
	a (=b)	c		Bi	Se	In	Sn
**Bi_2_Se_3_**	0.4142	2.8677	6.9236	4.78	12.7	0	0
**In-doped Bi_2_Se_3_**	0.4088	2.8767	7.0378	4.78	12.7	2.17	0
**Sn-doped Bi_2_Se_3_**	0.4140	2.8006	6.7641	4.78	12.7	0	2.11
**(In, Sn)-doped Bi_2_Se_3_**	0.4068	2.8719	7.0593	4.78	12.7	1.09	1.05

**Table 2:** Average diameter, average thickness, and FESEM–EDS of Bi, Se, In, and Sn (atomic percent, at.%) of pristine, and In-, Sn-, and (In, Sn)-doped Bi_2_Se_3_ nanoplatelets.

Sample	Average Diameter (μm)	Average Thickness (nm)	Bi (at.%)	Se (at.%)	In (at.%)	Sn (at.%)	Bi: Se
**Bi_2_Se_3_**	1.319	17.5	43.01	56.99	0.00	0.00	1:1.325
**In-doped Bi_2_Se_3_**	0.965	21.8	32.00	59.33	8.05	0.00	1:1.854
**Sn-doped Bi_2_Se_3_**	0.912	39.8	28.81	56.90	0.00	14.28	1:1.975
**(In, Sn)-doped Bi_2_Se_3_**	0.317	31.5	29.76	56.64	10.98	2.62	1:1.903

**Table 3:** The d-spacings and diffracted planes of pristine, and In-, Sn-, and (In, Sn)-doped Bi_2_Se_3_ nanoplatelets.

Sample	High-Resolution Images		SAD Patterns
	d-Spacings (nm)	Planes	Diffracted Planes
**Bi_2_Se_3_**	0.3470	(0 1 2)	(1 0 1)
	0.3225	(1 0 4)	(0 1 2)
			(1 0 4)
**In-doped Bi_2_Se_3_**	0.3404	(0 1 2)	(1 0 1)
	0.2958	(0 1 5)	(0 1 5)
			(0 1 11)
**Sn-doped Bi_2_Se_3_**	0.3011	(0 1 5)	(0 1 5)
			(1 0 7)
			(1 0 10)
			(1 1 0)
**(In, Sn)-doped Bi_2_Se_3_**	0.3546	(1 0 1)	(1 0 1)
	0.3096	(0 1 5)	(1 0 4)
			(0 1 11)
			(1 1 3)

**Table 4:** Characteristic Raman peaks of the pristine, and In-, Sn-, and (In, Sn)-doped Bi_2_Se_3_ nanoplatelets.

Sample	Raman Shift (cm^−1^)		
	A_1g_^1^	E_g_^2^	A_1g_^2^
**Bi_2_Se_3_**	87.21	133.21	176.36
**In-doped Bi_2_Se_3_**	82.65	131.10	174.43
**Sn-doped Bi_2_Se_3_**	82.02	130.54	171.90
**(In, Sn)-doped Bi_2_Se_3_**	81.93	133.64	177.48

**Table 5:** Details of photocurrent measurements of the I_max_, I_stable_, ΔI_decay_, τ_r_, t_decay_, τ_f_, and I_photo_/I_dark_ ratios under the UV light.

Sample	I_max_ (× 10^−10^ A)	I_stable_ (× 10^−10^ A)	ΔI_decay_ (× 10^−10^ A)	τ_r_ (sec)	t_decay_ (sec)	τ_f_ (sec)	I_photo_/I_dark_ (0 V Bias Voltage)
**Bi_2_Se_3_**	8.73	0.40	8.33	0.053	0.091	0.049	7.66
**In-doped Bi_2_Se_3_**	9.98	0.65	9.33	0.051	0.097	0.050	9.29
**Sn-doped Bi_2_Se_3_**	9.36	1.60	7.76	0.044	0.099	0.054	15.8
**(In, Sn)-doped** **Bi_2_Se_3_**	8.41	5.20	3.21	0.041	0.105	0.066	30.7

## Data Availability

Data are contained within the article.

## Supplementary Materials

The following are available online at https://www.mdpi.com/article/10.3390/nano11051352/s1, Figure S1: Schematic of the furnace, crucible, substrate, and operating conditions, Figure S2: Schematic diagram of the photocurrent measurement system, Figure S3: FESEM cross-sectional images of (**a**) pristine, (**b**) In-, (**c**) Sn-, and (**d**) (In,Sn)-doped Bi_2_Se_3_ nanoplatelets, Figure S4: Comparisons of the current in logarithmic scale vs. time under the (**a**) UV and (**b**) red light, Figure S5: FPA–FTIR absorbance and (**b**) estimated optical bandgaps of the pristine, and In-, Sn-, and (In,Sn)-doped Bi_2_Se_3_ nanoplatelets, Figure S6: The estimated optical bandgaps of (**a**) pristine, and (**b**) In-, (**c**) Sn-, and (**d**) (In,Sn)-doped Bi_2_Se_3_ nanoplatelets, Table S1: Lists of the Bi_2_Se_3_-based photodetectors, Table S2: Lists of the Bi_2_Se_3_ doped with various elements and the variation of the bandgap energy.
