# Supplementary material for: Photosensing and Characterizing of the Pristine and In-, Sn-Doped Bi2Se3 Nanoplatelets Fabricated by Thermal V–S Process
Source: Nanomaterials (Basel). 2021 May 20;11(5):1352. doi: 10.3390/nano11051352 (PMC8161412; doi:10.3390/nano11051352)
Supplement: Supplementary file 1 [file nanomaterials-11-01352-s001.zip › nanomaterials-1192832-supplementary.pdf]

Supplementary Materials

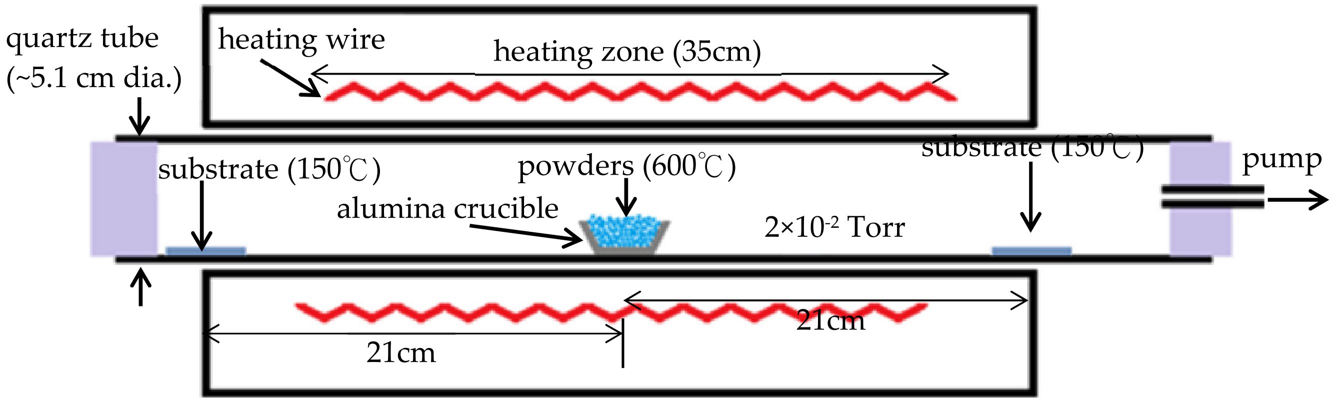

Figure S1. Schematic of the furnace, crucible, substrate and operating conditions.

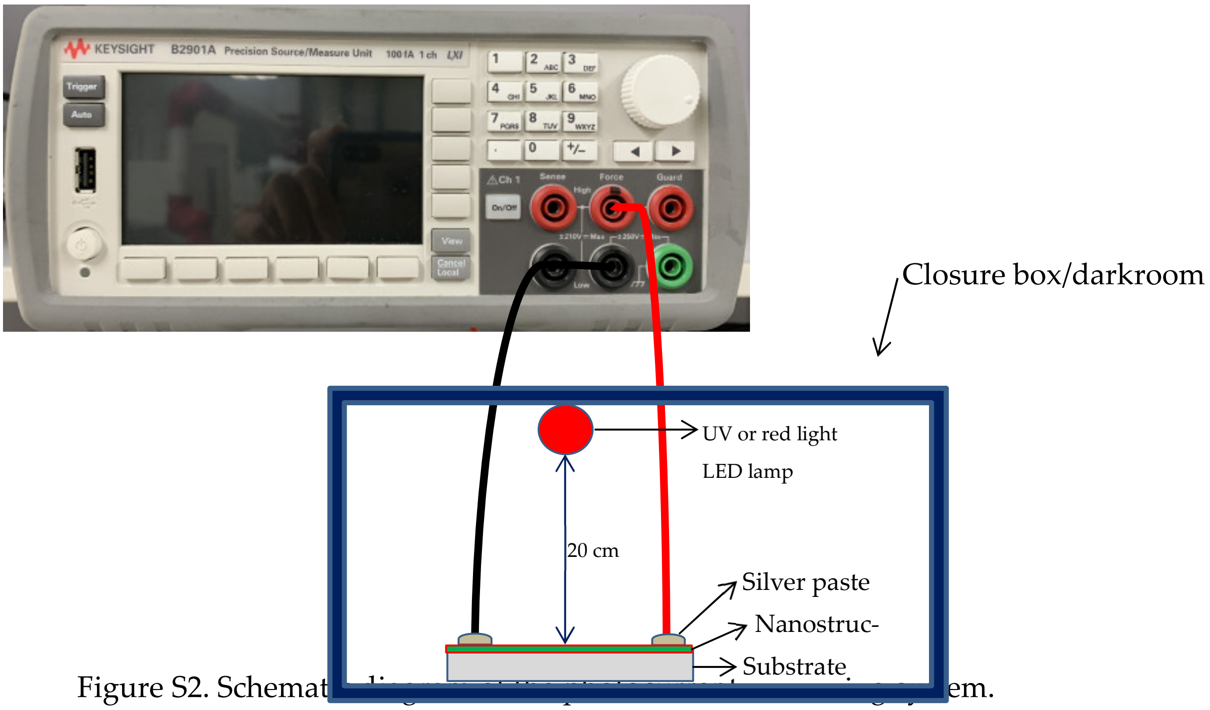

Figure S2. Schemat[illegible]em.

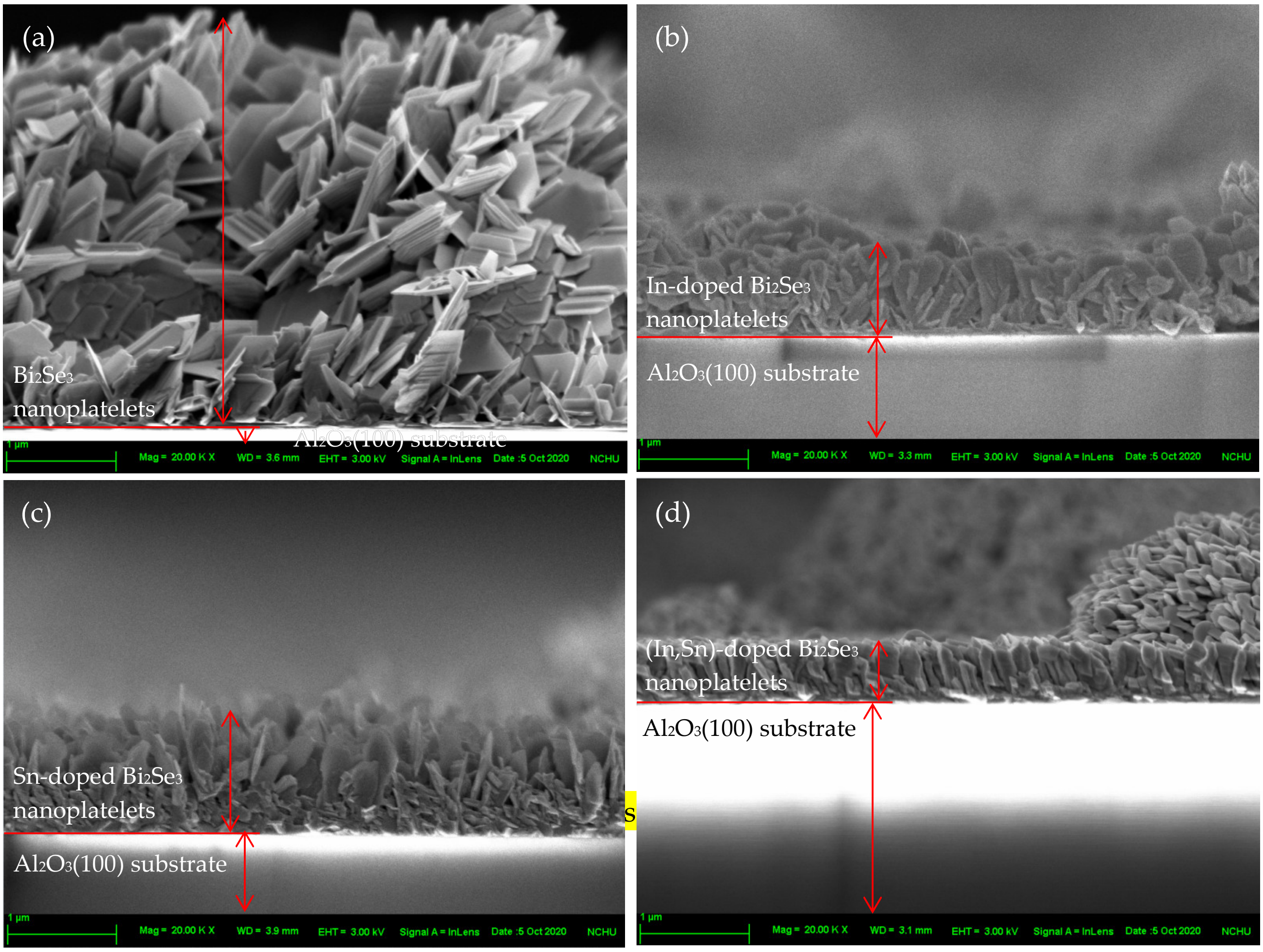
(a)
Bi2Se3
nanoplatelets
Al2O3(100) substrate
(b)
In-doped Bi2Se3
nanoplatelets
Al2O3(100) substrate
(c)
Sn-doped Bi2Se3
nanoplatelets
Al2O3(100) substrate
(d)
(In,Sn)-doped Bi2Se3
nanoplatelets
Al2O3(100) substrate

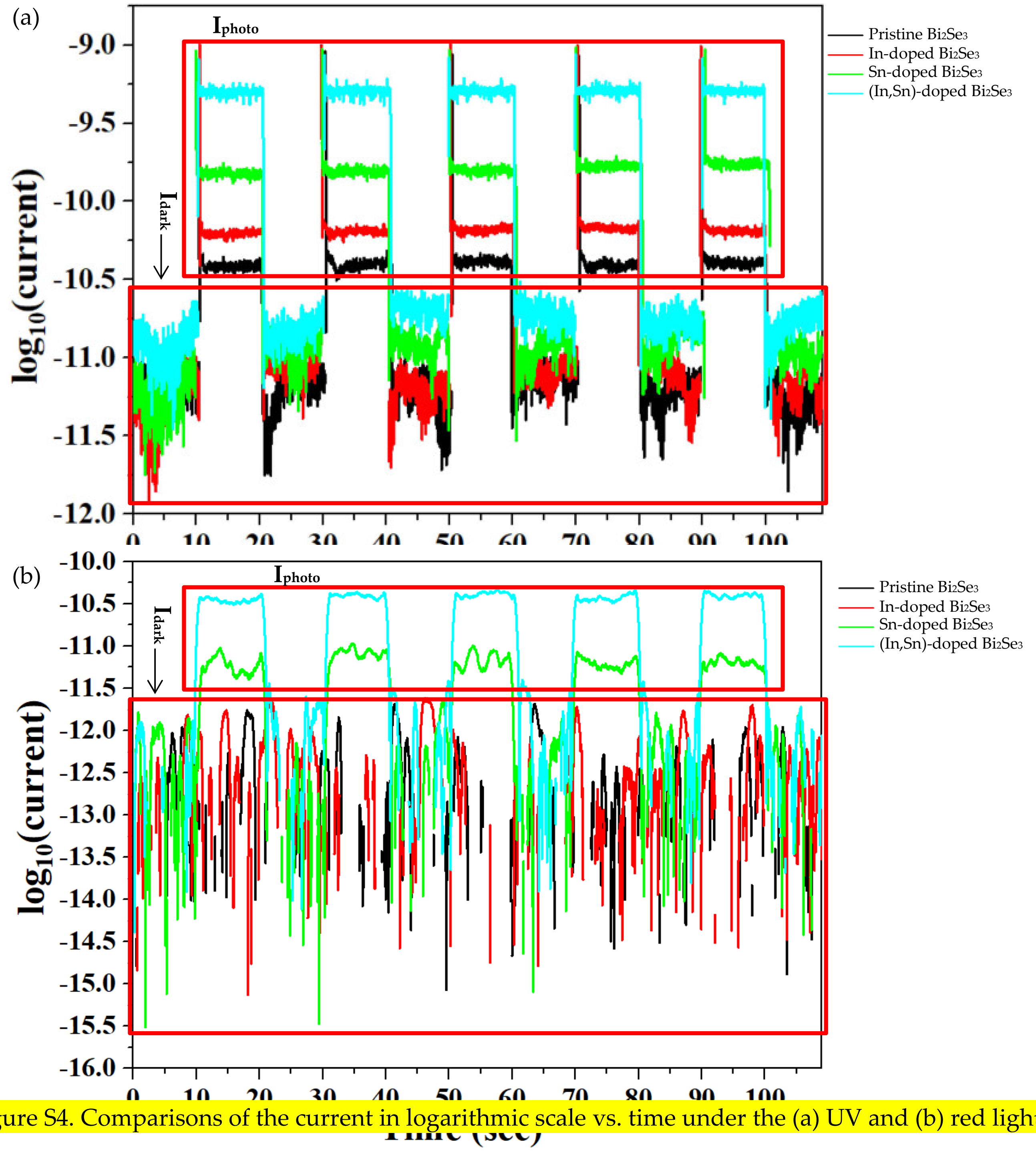

Figure S4. Comparisons of the current in logarithmic scale vs. time under the (a) UV and (b) red light.

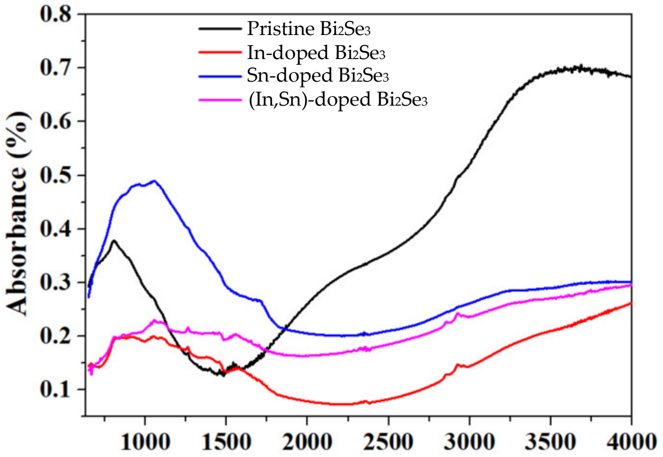

Figure S5. FPA-FTIR absorbance and (b) estimated optical band gaps of the pristine, and In-, Sn-, and (In,Sn)-doped Bi_2Se_3 nanoplatelets.

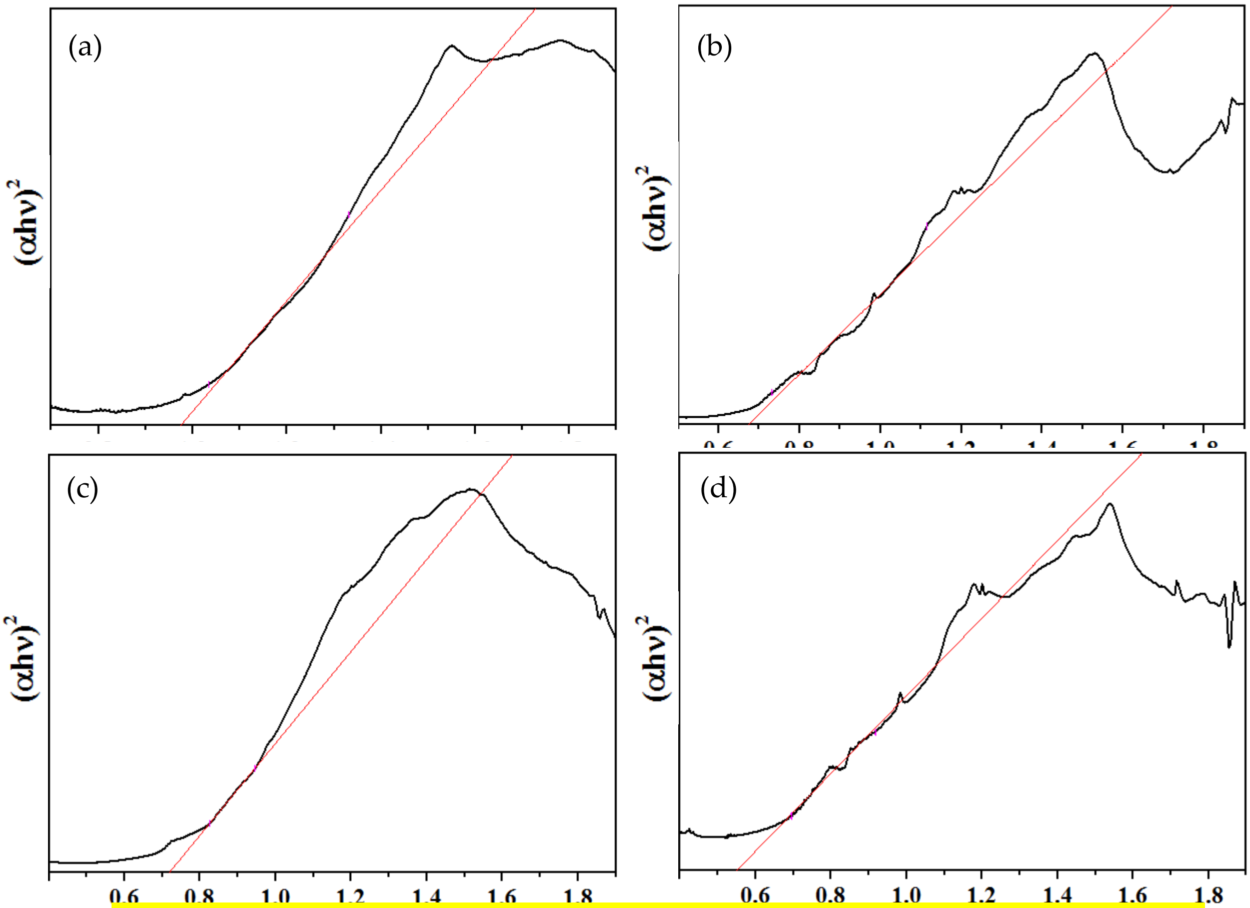

Figure S6. The estimated optical band gaps of (a) pristine, and (b) In-, (c) Sn-, and (d) (In,Sn)-doped Bi_2Se_3 nanoplatelets.

Table S1. Lists of the Bi_2Se_3-based photodetectors.

Materials	Substrate	Incident light wavelength (nm); power (mW/cm^2)	Incident light type	Reference
**Bi_2Se_3/MoO_3 thin films**	Mica	405–1550; 8.49	Laser	[1]
**Bi_2Se_3 nanowires/nanobelts**	Glass	460, 568, 595, 660, 1500; 0.25	LED	[2]
**Bi_2Se_3 nanowires**	Si(111)	785	Diode laser	[3]
**SnTe/Bi_2Se_3 thin films**	SiO_2/Si	1550; 0.16–9.33	Laser	[4]
**Bi_2Se_3 nanowires**	Si wafer	325, 442, 632, 808, 1060; 0–112.16	He-Cd laser/ micro laser	[5]
**Bi_2Se_3 flakes**	n/a	2.5×10^5 (0.12THz); 0.525	Laser	[6]
**Bi_2Se_3-$FA_{0.85}Cs_{0.15}PbI_3$-Bi_2Se_3 thin films**	Sapphire (0006)	365, 405, 520, 650, 808, 980; 0.0053–63.7	Diode laser	[7]
**Bi_2Se_3 nanoflakes/Si nanowires**	B-doped p-type Si (111)	890; 1–12.3	Xe lamp	[8]
**Graphene/ Bi_2Se_3 thin films**	Sapphire	3500; 0.05	Laser	[9]
**Bi_2Se_3 nanowires/SiO_2**	n-type Si wafer	808; 1.8–179.2	Laser diode	[10]
**Bi_2Se_3 thin films/ SiO_2**	n-type Si wafer	808; n/a<br>960; n/a<br>1310; 50–540<br>1550; 50–950	Laser	[11]
**Bi_2Se_3 nanowires/ SiO_2**	Si wafer	532; 32, 1064; 29	Laser	[12]
**Bi_2Se_3 nanoplatelets**	**Al_2O_3(100)**	365;8W, 700-900;5W	LED lamp	This work

Table S2. Lists of the Bi_2Se_3 doped with various elements and the variation of the band gap energy.

Bi_2Se_3 bulk band gap (eV)	Dopant	Doped Bi_2Se_3 Band gap (eV)	Reference
0.67	**Te**	0.7–0.78	[13]
0.30		0.06–0.20	[14]
1.73	**Sb**	1.80–1.94	[15]
n/a	**Mn**	n/a	[16]
0.114	**Pb**	0.105–0.127	[17]
0.32	**Dy**	0.42, 0.83	[18]
0.99	**Ni**	0.32	[19]
2.01		1.65	[20]
0.3	**Sn**	n/a	[21]
0.973		0.717	This work
0.32	**Cr**	0.01–0.28	[22]
0.32		0.01	[23]
n/a	**C**	n/a	[24]
n/a	**S**	n/a	[25]
n/a	**Eu**	n/a	[26]
n/a	**Cu**	n/a	[27]
n/a	**Ca**	n/a	[28]
n/a	**Nb**	n/a	[29]
n/a	**In**	n/a	[30]
0.973		0.641	This work
0.3	**V**	n/a	[31]
0.2–0.3	**Gd**	0.03	[32]
0.3	**Tl**	0.158	[33]
0.32	**Fe**	0.028	[22]
0.32		0.028–0.18	[21]

2.95	**Nd**	2.57–2.88	[34]
0.973	**Co-dopant of (In,Sn)**	0.548	This work

References

1. Yang, M.; Han, Q.; Liu, X.; Han, J.; Zhao, Y.; He, L.; Gou, J.; Wu, Z.; Wang, X.; Wang, J. Ultra-high stability 3D TI Bi_2Se_3/MoO_3 thin film heterojunction infrared photodetector at optical communication waveband. *Adv. Funct. Mater.* **2020**, 30, 1909659.
2. Salvato, M.; Scagliotti, M.; De Crescenzi, M.; Castrucci, P.; De Matteis, F.; Crivellari, M.; Cresi, S. P.; Catone, D.; Bauch, T.; F. Lombardi, Stoichiometric Bi_2Se_3 topological insulator ultra-thin films obtained through a new fabrication process for optoelectronic applications. *Nanoscale* **2020**, 12, 12405.
3. Meyer, N.; Geishendorf, K.; Walowski, J.; Thomas, A.; Münzenberg, M. Photocurrent measurements in topological insulator nanowires. *Appl. Phys. Lett.* **2020**, 116, 172402.
4. Zhang, H.; Song, Z.; Li, D.; Xu, Y.; Li, J.; Bai, C.; Man, B. Near-infrared photodetection based on topological insulator P-N heterojunction of $SnTe/Bi_2Se_3$. *Appl. Surf. Sci.* **2020**, 509, 145290.
5. Wang, X.; Dai, G.; Liu, B.; Zou, H.; Chen, Y.; Mo, X.; Li, X.; Sun, J.; Liu, Y.; Liu, Y.; Yang, J. Broadband photodetectors based on topological insulator Bi_2Se_3 nanowire with enhanced performance by strain modulation effect. *Physica E: Low-dimensional Systems and Nanostructures* **2019**, 114, 113620.
6. Tang, W.; Politano, A.; Guo, C.; Guo, W.; Liu, C.; Wang, L.; Chen, X.; Lu, W. Ultrasensitive Room-Temperature Terahertz Direct Detection Based on a Bismuth Selenide Topological Insulator. *Adv. Funct. Mater.* **2018**, 28, 1801786.
7. Liang, F. X.; Liang, L.; Zhao, X. Y.; Luo, L. B.; Liu, Y. H.; Tong, X. W.; Zhang, Z. X.; Huang, J. C. A. A sensitive broadband (UV–vis–NIR) perovskite photodetector using topological insulator as electrodes. *Adv. Optical Mater.* **2019**, 7, 1801392.
8. Das, B.; Das, N. S.; Sarkar, S.; Chatterjee, B. K.; Chattopadhyay, K. K. Topological insulator Bi_2Se_3/Si-nanowire-based p-n junction diode for high-performance near-infrared photodetector. *ACS Appl. Mater. Interfaces* **2017**, 9, 22788−22798
9. Kim, J.; Park, S.; Jang, H.; Koirala, N.; Lee, J. B.; Kim, U. J.; Lee, H. S.; Roh, Y. G.; Lee, H.; Sim, S.; Cha, S. ; In, C.; Park, J.; Lee, J.; Noh, M.; Moon, J.; Salehi, M.; Sung, J.; Chee, S. S.; Ham, M. H.; Jo, M. H.; Oh, S.; Ahn, J. H.; Hwang, S. W.; Kim, D.; Choi, H. Highly Sensitive, Gate-Tunable, Room-Temperature Mid-Infrared Photodetection Based on Graphene-Bi_2Se_3 Heterostructure. *ACS Photonics* **2017**, 4, 482−488.
10. Liu, C.; Zhang, H.; Sun, Z.; Ding, K.; Mao, J.; Shao, Z.; Jie, J. Topological insulator Bi_2Se_3 nanowire/Si heterostructure photodetectors with ultrahigh responsivity and broadband response. *J. Mater. Chem. C* **2016**, 4, 5648−5655.
11. Zhang, H.; Zhang, X.; Liu, C.; Lee, S. T.; Jie, J. High-Responsivity, High-detectivity, ultrafast topological insulator Bi_2Se_3/silicon heterostructure broadband photodetectors. *ACS Nano* **2016**, 10, 5113−5122.
12. Sharma, A.; Bhattacharyya, B.; Srivastava, A. K.; Senguttuvan, T. D.; Husale, S. High performance broadband photodetector using fabricated nanowires of bismuth selenide. *Sci.*

Rep. **2016**, 6, 19138.
13. Augustine, S.; Ampili, S.; Kang, J. K.; Mathai, E. Structural, electrical and optical properties of Bi_2Se_3 and $Bi_2Se_{(3x)}Te_x$ thin films. *Mater. Res. Bull.* **2005**, 40, 1314−1325.
14. Adam, A. M.; Elshafaie, A.; Mohamed, A. E. A.; Petkov, P.; Ibrahim, E. M. M. Thermoelectric properties of Te doped bulk Bi_2Se_3 system. *Mater. Res. Express* **2018**, 5, 035514.
15. Patil, N. S.; Sargar, A. M.; Mane, S. R.; Bhosale, P. N. Growth mechanism and characterisation of chemically grown Sb doped Bi_2Se_3 thin films. *Appl. Surf. Sci.* **2008**, 254, 5261−5265.
16. Sánchez-Barriga, J.; Varykhalov, A.; Springholz, G.; Steiner, H.; Kirchschlager, R.; Bauer, G.; Caha, O. ; Schierle, E.; Weschke, E.; Ünal, A. A.; Valencia, S.; Dunst, M.; Braun, J.; Ebert, H.; Minár, J.; Golias, E.; Yashina, L.V.; Ney, A.; Holy, V.; Rader, O. Nonmagnetic band gap at the Dirac point of the magnetic topological insulator $(Bi_{1-x}Mn_x)_2Se_3$, *Nat. Commun.* **2016**, 7, 10559.
17. Karamazov, S.; Horák, J.; Navrátil, J.; Lošták, P. Characterisation of Bi_2Se_3 crystals highly doped with Pb. *Cryst. Res. Technol.* **1997**, 32, 249−260.
18. Gupta, A.; Srivastava, S. K. Paramagnetism, hopping conduction, and weak localization in highly disordered pure and Dy-doped Bi_2Se_3 nanoplates. *J. Appl. Phys.* **2020**, 127, 244302.
19. Mohyedin, M. Z.; Malik, N. A.; Taib, M. F. M.; Mustaffa, M.; Hassan, O. H.; Ali, A. M. M.; Haq, B. U.; Yahya, M. Z. A. First principles study of structural, electronic and optical properties of orthorhombic phase Ni-doped Bi_2Se_3 using density functional theory. *Comput. Condens. Matter* **2020**, 25, e00510.
20. Mazumder, K.; Sharma, A.; Kumar, Y.; Shirage, P. M. Temperature dependent I-V characteristics of Ni doped topological insulator Bi_2Se_3 nanoparticles. *AIP Conference Proceedings* **2019**, 2115, 030147.
21. Stephen, G. M.; Naumov, I.; Tyagi, S.; Vail, O. A.; DeMell, J. E.; Dreyer, M.; Butera, R. E.; Hanbicki, A. T.; Taylor, P. J.; Mayergoyz, I.; Dev, P.; Friedman, A. L. Effect of Sn doping on surface states of Bi_2Se_3 thin films. *J. Phys. Chem. C* **2020**, 124, 27082−27088.
22. Zhang, J. M.; Zhu, W.; Zhang, Y.; Xiao, D.; Yao, Y. Tailoring magnetic doping in the topological
insulator Bi_2Se_3. *Phys. Rev. Lett.* **2012**, 109, 266405.
23. Zhang, J. M.; Ming, W.; Huang, Z.; Liu, G. B.; Kou, X.; Fan, Y.; Wang, K. L.; Yao, Y. Stability, electronic, and magnetic properties of the magnetically doped topological insulators Bi_2Se_3, Bi_2Te_3, and Sb_2Te_3. *Phys. Rev. B* **2013**, 88, 235131.
24. Xin, X.; Guo, C.; Pang, R.; Zhang, M.; Shi, X.; Yang, X.; Zhao, Y. Theoretical and experimental studies of spin polarized carbon doped Bi_2Se_3. *Appl. Phys. Lett.* **2019**, 115, 042401.
25. Jin, W.; Wang, Z. Sulfur-doped Bi_2Se_3 monolayer as potential electrode materials for Mg-ion batteries. *IOP Conf. Ser.: Mater. Sci. Eng.* **2019**, 677, 022125.
26. Sultana, R.; Gurjar, G.; Gahtori, B.; Patnaik, S.; Awana, V. P. S. Structural, surface morphology and magneto-transport properties of self flux grown Eu doped Bi_2Se_3 single crystal. *Mater. Res. Express* **2019**, 6, 096107.
27. Li, M.; Wang, Z.; Yang, L.; Gao, X. P. A.; Zhang, Z. From linear magnetoresistance to parabolic magnetoresistance in Cu and Cr-doped topological insulator Bi_2Se_3 films. *J. Phys. Chem. Solids* **2019**, 128, 331−336.
28. Zhang, M.; Liu, L. G.; Wang, D.; An, X. Y.; Yang, H. Enhancement of surface state contribution

in cadmium doped Bi_2Se_3 single crystal. *J. Alloys Compd.* **2019**, 806, 180−186.
29. Kurter, C.; Finck, A. D. K.; Huemiller, E. D.; Medvedeva, J.; Weis, A.; Atkinson, J. M.; Qiu, Y.; Shen, L.; Lee, S. H.; Vojta, T.; Ghaemi, P.; Hor, Y. S.; Van Harlingen, D. J. Conductance spectroscopy of exfoliated thin flakes of $Nb_xBi_2Se_3$. *Nano Lett.* **2019**, 19, 38−45.
30. Kimura, K.; Hayashi, K.; Yashina, L. V.; Happo, N.; Nishioka, T.; Yamamoto, Y.; Ebisu, Y.; Ozaki, T. ; Hosokawa, S.; Matsushita, T.; Tajiri, H. Local structural analysis of In-doped Bi_2Se_3 topological insulator using X-ray fluorescence holography. *Surf. Interface Anal.* **2019**, 51, 51−55.
31. Zhang, L.; Zhao, D.; Zang, Y.; Yuan, Y.; Jiang, G.; Liao, M.; Zhang, D.; He, K.; Ma, X.; Xue, Q. Ferromagnetism in vanadium-doped Bi_2Se_3 topological insulator films. *APL Mater.* **2017**, 5, 076106.
32. Zheng, F.; Zhang, Q.; Meng, Q.; Wang, B.; Fan, L.; Zhu, L.; Song, F.; Wang, G. Electronic structures and magnetic properties of rare-earth (Sm, Gd) doped Bi_2Se_3. *Chalcogenide Lett.* **2017**, 14, 551−560.
33. Saeed, Y.; Singh, N.; Schwingenschlögl, U. Enhanced thermoelectric figure of merit in strained Tl-doped Bi_2Se_3. *Appl. Phys. Lett.* **2014**, 105, 031915.
34. Alemi, A.; Babalou, A.; Dolatyari, M.; Klein, A.; Meyer, G. Hydrothermal synthesis of Nd^{III} doped Bi_2Se_3 nanoflowers and their physical properties. *Z. Anorg. Allg. Chem.* **2009**, 635, 2053−2057.
